# The Royal Statistical Society's National Value

**Published:** 1917-12-01

**Authors:** 


The Hospital

The Workers' Newspaper of Administrative Medicine and Institutional Life, Administration, National Insurance and Health.

No. 1643, Vol. LXIII. SATURDAY, DECEMBER 1, 1917. PRICE ONE PENNY.

### THE ROYAL STATISTICAL SOCIETY'S NATIONAL VALUE.

Statistical records may seem cold and lifeless to the uninitiated, but such is by no means the view of the born or developed statistician. For him figures and their meaning are the food of the mind, and not very infrequently he is able out of such apparently unpromising material to develop a large measure of controversial zeal. There are, it appears, more ways of using figures than the plain man suspects, and their meanings do not invariably lie on the surface. The short way of dismissing them as more untrustworthy even than facts only shows that they need skilled interpretation. The statistical conjuror may, either by intention or ignorance, produce truly surprising deductions, but this does not mean that the statistical expert is to be mistrusted. Of this latter authority something has been heard in recent years even among the statistical records of the medical profession, and many of these and the conclusions founded on them have received somewhat scant courtesy. There is, it seems, an inner grace in these things, and not all the medical writers have acquired it. Not that the statistician has had it all his own way. On the contrary, his attack has been vigorously challenged, and more particularly by Sir Almroth Wright, who has expounded a philosophy of figures which seems rank heresy to the orthodox statistical creed. Medicine and statistics have in a few instances married one another, and a fateful issue may result from the combination.

A recent presidential address to the Royal Statistical Society by Sir Bernard Mallet impresses upon us the important part played by the Society in the promotion of the love of figures in our midst. There is, it seems, no Government central statistical office. Each official department compiles and arranges its own numerical records, and we gather from the President that some of them employ methods that are at least open to discussion. In the very nature of things the officials concerned must be inclined to place their figures in the most favourable light, for by these is set forth the measure of the department's achievement. Everyone must allow that there is an advantage in the existence of an apparatus capable of independent and impartial examination, and this function the Statistical Society endeavours to discharge. The war has provided difficulties, but it has also provided material, for out of it have arisen influences which modify seriously the vital statistics of the nations, and these have a large bearing both on present and future policy. Hence even those of us who stand without may contemplate the work of the Statistical Society with interest and good will, and may be thankful that it verifies and cultivates with confidence facts which are of high national importance.

As illustrating the value and significance of the Society's enterprise, we may refer in general terms to some of the statistical consequences of the war as these are displayed in detail in the President's address, and it will be noted that most of these have a close relation to public policy. In this connection the figures showing the great decline in the deaths certified as due to alcohol are very impressive, seeing that in this direction there has been a special degree of interference with the national habits. It has often been said that people cannot be made sober by Act of Parliament, but here we have a plain intimation that restricted opportunity means lessened abuse, and it is difficult to explain the figures in any other fashion. There is a somewhat odd confirmation of this conclusion in the figures relative to the deaths of children overlain by their parents. It is generally allowed that intoxication or semi-intoxication is a large factor in these catastrophes, and one of the experiences supporting this view is that such accidents used to be particularly frequent on Saturday nights. But the figures for 1916, as compared with those for 1913, show a decline in such deaths of nearly 50 per cent., and though Saturday night still shows its bad eminence this has been reduced in even a greater proportion than the general record. There have thus been fewer deaths from alcohol and fewer also of infants suffocated while sleeping with their parents, and both announcements point to one and the same conclusion. In some measure, doubtless, is to be attributed to the same source a decided decline in the numbers of suicides, though probably a diminution of unemployment here plays some part.

On the question of the total population it is calculated that three years of war have allowed some slight increase in the numbers in the United Kingdom, and that in this respect we are far better off than are the principal enemy countries, and this apart from the heavier losses which these countries have suffered on the battlefield. Two minor items of interest in connection with births relate to illegitimacy and the proportions of the sexes. Regarding the former it will be remembered there were in early days some highly sensational rumours in circulation. What do the actual figures say? They show conclusively that the story was a newspaper boom, for there has been no appreciable increase in the numbers of illegitimate births, and, indeed, these in 1915 were the lowest on record. The point may be taken as an example of the value of statistical studies. Yet out of such studies unanimity is not an invariable result. Thus on the influence of war on the sex ratio at birth, while some have affirmed an accentuation of the excess of males over females others have denied any such experience. In the figures for the United Kingdom for 1915 and 1916 there is certainly support for the assertion, for the totals show 1,046 males to 1,000 females, and this gives to the males eight above the average for the last forty years. These few quotations give at least a hint of the interest of the work prosecuted by the Royal Statistical Society, and those of our readers who favour such studies should certainly read the full text of the President's address.

